# SMOC2 inhibits calcification of osteoprogenitor and endothelial cells

**DOI:** 10.1371/journal.pone.0198104

**Published:** 2018-06-13

**Authors:** Tine Peeters, Silvia Monteagudo, Przemko Tylzanowski, Frank P. Luyten, Rik Lories, Frédéric Cailotto

**Affiliations:** 1 Laboratory of Tissue Homeostasis and Disease, Skeletal Biology and Engineering Research Center, Department of Development and Regeneration, KU Leuven, Leuven, Belgium; 2 Laboratory for Developmental and Stem Cell Biology, Skeletal Biology and Engineering Research Center, Department of Development and Regeneration, KU Leuven, Leuven, Belgium; 3 Department of Biochemistry and Molecular Biology, Medical University of Lublin, Lublin, Poland; 4 Division of Rheumatology, University Hospitals Leuven, Leuven, Belgium; 5 UMR 7365 CNRS‐Université de Lorraine, Ingénierie Moléculaire et Physiopathologie Articulaire (IMoPA), Biopôle de l’Université de Lorraine, Campus Biologie-Santé, Vandoeuvre Les Nancy, France; University of Texas Southwestern Medical Center, UNITED STATES

## Abstract

Tissue calcification is an important physiological process required for the normal structure and function of bone. However, ectopic or excessive calcification contributes to diseases such as chondrocalcinosis, to calcium deposits in the skin or to vascular calcification. SMOC2 is a member of the BM-40/osteonectin family of calcium-binding secreted matricellular proteins. Using osteoprogenitor MC3T3-E1 cells stably overexpressing SMOC2, we show that SMOC2 inhibits osteogenic differentiation and extracellular matrix mineralization. Stable *Smoc2* knockdown in these cells had no effect on mineralization suggesting that endogenous SMOC2 is not essential for the mineralization process. Mineralization in MC3T3-E1 cells overexpressing mutant SMOC2 lacking the extracellular calcium-binding domain was significantly increased compared to cells overexpressing full length SMOC2. When SMOC2 overexpressing cells were cultured in the presence of extracellular calcium supplementation, SMOC2’s inhibitory effect on calcification was rescued. Our observations were translationally validated in primary human periosteal-derived cells. Furthermore, SMOC2 was able to impair mineralization in transdifferentiated human umbilical vein endothelial cells. Taken together, our data indicate that SMOC2 can act as an inhibitor of mineralization. We propose a possible role for SMOC2 to prevent calcification disorders.

## Introduction

Tissue calcification is an important and physiological process required for the normal structure and function of bone [1]. Calcification of the bone extracellular matrix gives the bone and body structure, helps to protect the inner organs and is a storage site from which calcium can be mobilized when required. However, abnormal or excessive calcification of tissues contributes to symptoms or complications of different diseases. For instance, chondrocalcinosis is a skeletal disorder in which calcium pyrophosphate crystals are deposited in the joints and tendons, triggering acute and painful inflammation [2]. Moreover, calcium crystal deposits occur in the skin in patients suffering from systemic sclerosis. Also, calcium crystal deposits can be found in arteries, a feature associated with increased cardiovascular risk. Vascular calcification most often occurs in patients suffering from diabetes, renal insufficiency or atherosclerosis [3–5]. Thus, there is need for effective strategies that prevent pathological calcification.

SMOC2 (SPARC-related modular calcium-binding protein 2) is a secreted calcium-binding protein from the BM-40/SPARC/osteonectin family of secreted matricellular proteins. BM-40/SPARC/osteonectin family members all contain an extracellular calcium-binding (EC) domain, a follistatin-like (FS) domain and an acidic N-terminal domain. SMOC2 has a unique composition different from the other family members as 2 thyroglobulin domains and a SMOC-specific domain separate the EC domain and FS domain [6–8].

SMOC2 was originally identified from an extracellular extract of the articular cartilage [9–11], a tissue in which calcification must be avoided. Indeed, the uncalcified proteoglycan and water rich extracellular matrix of the articular cartilage allows efficient and low-friction mobility between the bones. This function must be preserved during aging to avoid the development of osteoarthritis, the most common chronic joint disease [12]. Based on its structure and its expression in the articular cartilage, we hypothesized that SMOC2 may have inhibitory effects on calcification. Thus, we investigated the effect of SMOC2 on mineralization and calcification. We demonstrate, in different *in vitro* models, that SMOC2 strongly inhibits calcification. Calcium sequestration by SMOC2’s calcium binding domain is proposed as part of the underlying mechanism.

## Materials and methods

### Materials and cells

All products used were purchased from Sigma unless otherwise stated. Human periosteum-derived cells (hPDC) and human umbilical vein endothelial cells (HUVEC) were a kind gift of the Tissue Engineering Unit, SBE center, KU Leuven. All procedures were approved by the ethical committee for clinical research (UZ Leuven), and informed consent was obtained from the patients.

### Generation of stable gene overexpression or silencing cell lines

MC3T3-E1 cells were plated at a density of 2,600 cells/cm^2^ in a 6 well-plate and transfected with 2 μg of an empty pcDNA3.1+ vector (3.1) as a control, the pcDNA3.1-*Smoc2* (*Smoc2+*), pcDNA3.1-*Smoc2* lacking the calcium binding domain (ΔCaBD), non-interfering short hairpin micro (shmi)RNA (Gipz) or a shmiRNA against *Smoc2* (Sh*Smoc2*). pcDNA3.1-*Smoc2* ΔCaBD was generated by performing PCR-directed mutagenesis using the pcDNA3.1-*Smoc2* as a template as explained in the scheme in S1 Fig. Briefly, the calcium binding domain spans from aminoacid 352 to 412. For the first PCR reaction, we used the pcDNA3.1 plasmid containing wild type *Smoc2* and primer pair A (P1 and P2) to obtain the PCR product A and primer pair B (P3 and P4) to obtain PCR product B. Primers were designed in such a way that the products had an overlap to bind to each other when used as templates in PCR reaction 2. The resulting product is the pcDNA3.1 plasmid containing mutant *Smoc2* lacking the calcium binding domain (ΔAA352-412). Primers sets were manually designed using free internet software Primer3 (http://frodo.wi.mit.edu/primer3/). Primer set A consisted of P1 fwd, 5’-ATGCTGCCGCCACAGCTGT-3’; and P2 rev, 5’-GACACCCAAAACCCTCTCCTCCAGGGTGTG -3’. Primer set B consisted of P3 fwd, 5’-GAGAGGGTTTTGGGTGTCACCAGAGAGGAG-3’; and P4 rev, 5’-TCATCCTTGTTTCCTGGGCTGT-3’. ShmiRNA against *Smoc2* was purchased from Open Biosystems (now Dharmacon; clone ID V2LMM_74468). The transfection reagent Arrest-In (Thermo Scientific) was used for the transfection reaction. To select stable lines, cells were treated for 10 days with the appropriate selection antibiotics (3.1, *Smoc2+*, *Smoc2* ΔCaBD at 1 mg/ml of geneticin, (G418) Gibco; Gipz, Sh*Smoc2* at 0.5 μg/μl of puromycin (Life technologies/invitrogen)). Then, single colonies were created with a dilution method in which a single resistant cell was isolated by serial half-dilutions. The single cell was then cultured and expanded keeping the appropriate antibiotic pressure.

### In vitro experiments

#### MC3T3-E1 cells

MC3T3-E1 cells stably transfected with either the empty pcDNA3.1+ vector or with the pcDNA3.1-*Smoc2* (mouse) overexpression vector were cultured in Dulbecco’s modified Eagle’s medium (DMEM) (2 mM Glutamax; 4.5 g/L high glucose, Gibco) supplemented with 10% fetal bovine serum (FBS, Gibco), 1% sodium pyruvate (Gibco), 1% antibiotics-antimycotic (penicillin-streptomycin/amphotericin B, Gibco). Cells were seeded at 2600 cells/cm^2^ in 6-well plates (D0) and induction of MC3T3-E1 mineralization started the day after (D1) by culturing cells for 21 days in Alpha Minimal Essential medium (αMEM) from Gibco, supplemented with 10% FBS, 1% sodium pyruvate, 1% antibiotics-antimycotic, 10 mM β-glycerophosphate (β-GP) and 50 μg/ml ascorbic acid-2-phosphate (AA). D1 samples represent undifferentiated cells whereas D21 samples are differentiated. Medium was changed every 2 days, but AA was added daily. The suitable antibiotic pressure (used to establish the stable clones) was maintained for the duration of the experiment using either 1 mg/ml of G418 or 0.5 μg /ml of puromycin.

#### Human periosteum-derived cells (hPDCs) and human umbilical vein endothelial cells (HUVECs)

Human periosteum-derived cells (hPDCs) were seeded at a density of 4,500 cells/cm^2^. Human umbilical vein endothelial cells (HUVECs) were seeded on gelatin-coated plastic at a density of 4,500 cells/cm^2^ and stimulated in basal endothelial cell growth (EGM)-2 medium (Lonza) in the presence of 200 ng/ml of Bone morphogenetic protein 6 (BMP6; R&D systems), known to trigger the endothelial to mesenchymal transition [13]. Both hPDCs and HUVECs were then cultured for 21 days as MC3T3-E1 cells but supplemented with 100 nM dexamethasone to trigger calcification. hPDCs and HUVECs were cultured in the presence of supernatants harvested from either 3.1, *Smoc2+* or ΔCaBD MC3T3-E1 cells.

### Alizarin red staining

At D1 and D21, samples were washed with phosphate buffered saline (PBS) and fixed with ice-cold methanol for at least 30 min at 4°C. Cells were then washed again with PBS and stored in water at 4°C until further use. Plates were stained for 1 hour with 1% Alizarin red solution pH 4.1–4.3 and washed with water afterwards. The staining was then quantified using a protocol according to Gregory C.A. et al [14].

### Gene expression analysis

At D1 and D21, samples were washed with PBS and stored dry at -80°C until further use. Total RNA was extracted using the NucleoSpin RNA II kit (Macherey-Nagel). Gene expression was analyzed by RT-qPCR using Maxima SYBR Green qPCR Master Mix (Fermentas) in the rotor-gene 6000 (Corbett research). The ΔΔCt method was used to determine fold changes. 40S ribosomal protein *S29* was used as internal control gene for normalization. ΔCt values are calculated as Ct (gene of interest)–Ct (S29). Fold change for each gene of interest compared to reference (control D1) was equal to 2^-ΔΔCt^ with ΔΔCt = ΔCt (target)—ΔCt (reference). Mouse *S29* was detected using the following primers: *S29* fwd, 5’-CCAGCAGCTCTACTGGAGTCA-3’; and *S29* rev, 5’-GCCTATGTCCTTCGCGTACT-3’. For endogenous mouse *Smoc2*, the following primers were used: *Smoc2* fwd, 5’-CAAGTGCAAAGATCCACAGC-3’; and *Smoc2* rev, 5’-GCCTATGTCCTTCGCGTACT-3’. Osteogenic markers mouse *Osx*, *Opn* and *Col1a2* were detected by using the following primers: *Osx* fwd, 5’-ATGGCGTCCTCTCTGCTTGA-3’; and *Osx* rev, 5’-AGTCCCGCAGAGGGCTAGAG-3’; *Opn* fwd, 5’-TCCAATCGTCCCTACAGTCG-3’; and *Opn* Rev, 5’-AGGTCCTCATCTGTGGCATC-3’; *Col1a2* fwd, 5’-GGCAGAGATGGTGTTGATGG-3’; and *Col1a2* rev, 5’-AGGGCCAGATGAAACTCCTT-3’.

### Western blot analysis

Total protein was extracted by adding 0.05% TritonX-100 to the samples followed by ultrasonication (Microson ultrasonic cell disruptor, Misonic). Afterwards, half of the samples were stored at -80°C for alkaline phosphatase (ALP) assay. Phosphatase inhibitors Na_3_VO_4_ 2.3 mM and 5 mM and protease inhibitor phenylmethylsulfonyl fluoride (PMSF) 0.36 mM and the commercial protease inhibitor cocktail PIC (Sigma P2714)) were added to the other half of the samples. Proteins were quantified according to the Pierce bicinchoninic acid (BCA) Protein assay kit (Thermo scientific). Absorbance was measured with the micro plate reader Titertek plus MS212 at 570 nm. For Western blot analysis, supernatants from MC3T3-E1 cells were harvested and clarified by centrifugation at 12,000 × *g* for 5 min. The same volume of supernatants (10 μl for each sample) was placed in a final concentration of 1× Laemmli buffer. Proteins were separated by electrophoresis on a NuPAGE® Bis-Tris gel (Invitrogen) and transferred from the gel to an activated polyvinylidene difluoride (PVDF) membrane (Millipore) using the Trans-blot® SD semi-dry electrophoretic transfer cell system (Biorad). Ponceau red staining was performed to confirm equal protein loading. Membranes were blocked with 5% non-fat dry milk in Tris buffered saline with 0,1% Tween (TBS-T). Mouse SMOC2 was detected with anti-SMOC2 rabbit polyclonal (diluted 1:1000; Santa Cruz sc-67396 (no longer available)), followed by incubation with anti-rabbit horseradish peroxidase-linked secondary antibody (diluted 1:5000; Jackson ImmunoResearch). The target protein was visualized using the Supersignal West Femto maximum Sensitivity Substrate kit (Thermo Scientific) and the Las3000 mini (Fujifilm).

### Alkaline phosphatase assay

An ALP assay was performed using the BluePhos Microwell Substrate kit (KPL). Briefly, 20 μl of proteins (stored at -80°C) were placed, in duplicate, in a 96-well plate. 50 μl of work solution (mix of ½ solution A and ½ solution B) was added to every sample followed by incubation at 37°C. Absorbance at 620 nm was measured after 15 minutes of incubation and results were expressed as absorbance values normalized to total protein content.

### Statistical analysis

Results are presented as the mean ± standard deviation (SD) of three independent replicates, unless otherwise specified. Comparisons were made by 2- way ANOVA using GraphPad Prism 7 software. A value of *P* < 0.05 was considered significant.

## Results

### SMOC2 inhibits osteogenic differentiation and ECM mineralization

**Fig 1:**
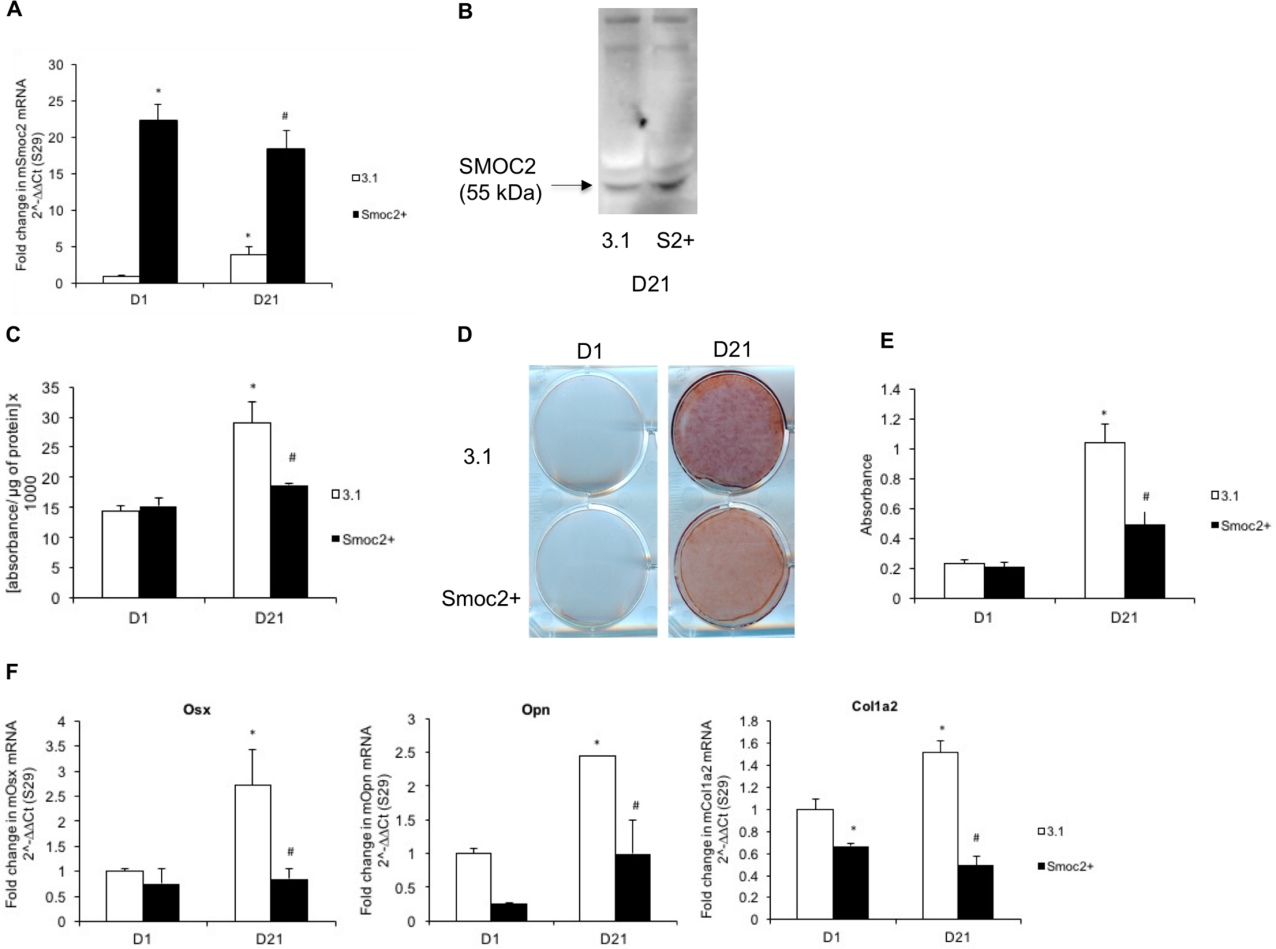
Influence of full length *Smoc2* overexpression on differentiation and mineralization of MC3T3-E1 cells. Gene expression (A) and Western blot (B) analysis of MC3T3-E1 cells overexpressing *Smoc2* show successful overexpression of *Smoc2*. At D21 *Smoc2+* cells show smaller increase in ALP activity (C) and less Alizarin red staining (D-E) compared to 3.1. (F) *Smoc2+* modifies mRNA expression of *Opn*, *Osx* and *Col1a2* during osteogenic differentiation. Statistically significant differences vs. D1 3.1 (internal control) are indicated as *: *P*<0.05; vs. D21 3.1 as #: *P*<0.05). Figures are representative of two independent experiments with three different *Smoc2+* overexpressing clones, each experiment performed in triplicates. mRNA levels were normalized to *S29*.

To study how SMOC2 affects osteogenic differentiation and mineralization, we overexpressed *Smoc2* in MC3T3-E1 osteoblast precursor cells (*Smoc2+*) and differentiated these into mature osteoblasts that produce a calcified extracellular matrix (ECM). Following stable transfection, relative *Smoc2* mRNA levels were determined in *Smoc2+* cells compared to control cells expressing an empty vector (3.1) at baseline (day 1) and after 21 days in the differentiation culture. At day 1, *Smoc2* mRNA levels were highly upregulated (20-fold increase) in *Smoc2+* cells compared to 3.1 cells (Fig 1A). This upregulation was sustained during the differentiation process. In control cells, endogenous *Smoc2* expression was increased after osteogenic differentiation but remained about 4 times lower than in *Smoc2+* cells. We then assessed the secretion of SMOC2 protein in supernatants of differentiated *Smoc2+* cells compared to 3.1+ cells by Western blot. These results confirm that secreted SMOC2 levels from the modified cells remain increased after 21 days of culture (Fig 1B).

As a functional readout for osteogenic differentiation, ALP activity was measured (Fig 1C). At baseline, no difference in ALP activity could be detected between *Smoc2+* and control cells. However, the expected increase in ALP levels after differentiation at day 21 was significantly lower in Smoc2+ cells compared to control cells. As a marker for matrix mineralization, calcium deposition was evaluated by alizarin red staining. At baseline, we did not detect mineralization in *Smoc2+* or control cells, but after 21 days of differentiation, matrix mineralization was significantly lower in *Smoc2+* cells than in control cells (Fig 1D and 1E). Messenger RNA levels of phenotypic markers of osteogenic differentiation, i.e. transcription factor *osterix* (*Osx)*, ECM gene *osteopontin* (*Opn)* and bone-related *collagen 1a2* (*Col1a2)* were assessed by RT-q-PCR. At day 1, no significant difference could be detected in mRNA expression level of *Osx and Opn*, whereas *Col1a2* was significantly decreased in *Smoc2+* cells compared to control cells. The increase in mRNA levels of *Osx* and *Col1a2* over time after 21 days of differentiation was significantly lower in *Smoc2+* cells compared to control cells. *Smoc2+* cells showed significantly reduced *Opn* mRNA levels at D21 (Fig 1F). Altogether, these data support that SMOC2 acts as negative modulator of osteogenic differentiation and ECM mineralization in MC3T3-E1 cells.

### SMOC2 is not essential for osteogenic differentiation and ECM mineralization

**Fig 2:**
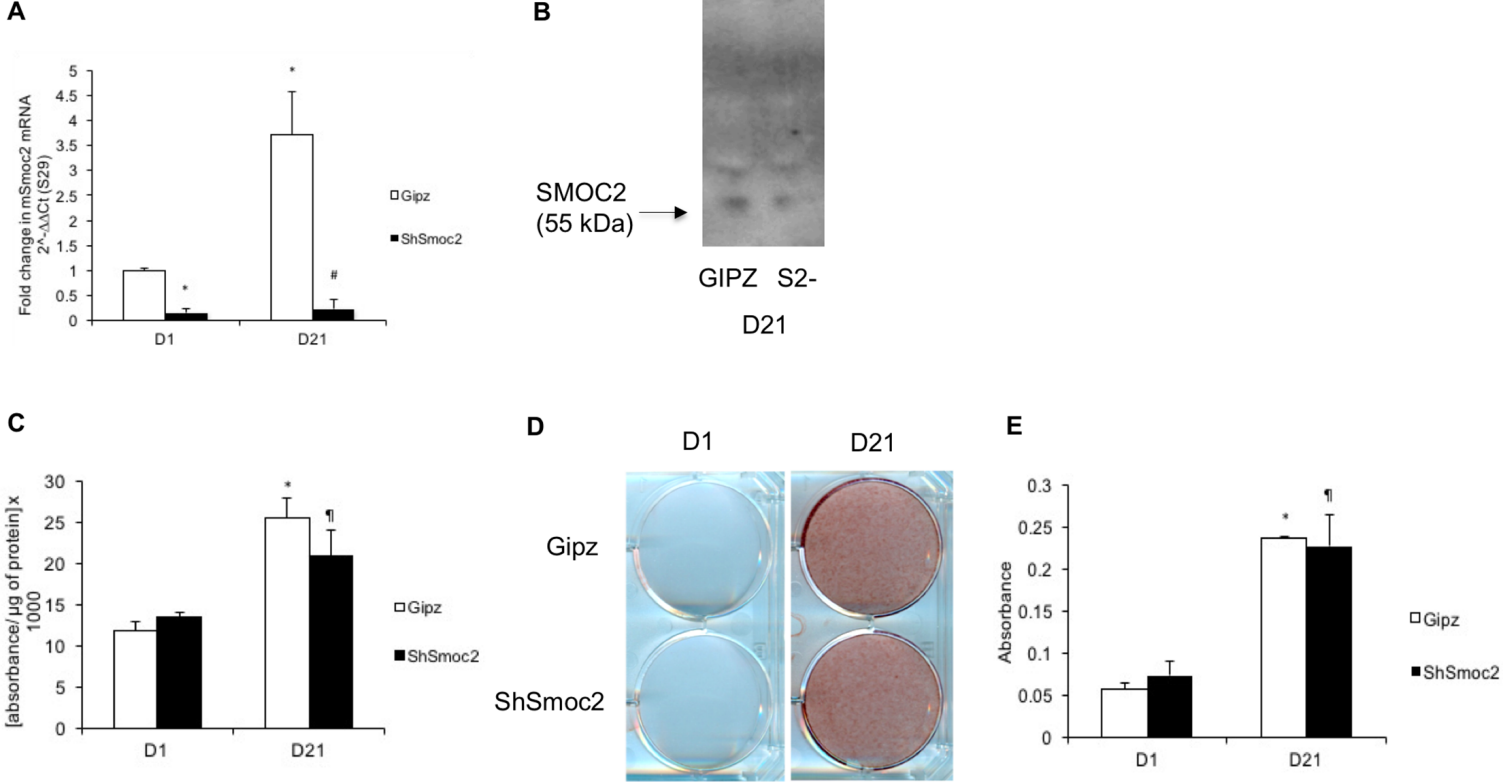
Influence of full length *Smoc2* knock down on differentiation and mineralization of MC3T3-E1 cells. Gene expression (A) and western blot (B) analysis of MC3T3-E1 cells with *Smoc2* knock down show successful knockdown of *Smoc2*. At D21 Sh*Smoc2* cells show no difference in ALP activity (C) and Alizarin red staining (D-E) compared to control cells (Gipz). Statistically significant differences vs. D1 Gipz (internal control) are indicated as *: *P*<0.05; vs. Sh*Smoc2* at D1 as ¶: *P*<0.05; vs. D21 Gipz as #: *P*<0.05). Figures are representative of 3 independent experiments with 3 different Sh*Smoc2* clones, each experiment performed in triplicate.

To further examine the role of SMOC2 in osteogenic differentiation and mineralization *in vitro*, we generated MC3T3-E1 cells in which endogenous *Smoc2* was silenced. Relative *Smoc2* mRNA levels were determined in control cells expressing a non-interfering short hairpin micro (shmi)RNA (Gipz) and cells with stable knock-down generated by shmiRNA against *Smoc2* (Sh*Smoc2*), after 1 and 21 days in culture. Fig 2A confirms lower *Smoc2* mRNA level in Sh*Smoc2* cells compared to control cells both at D1 and D21 (6-fold and 16-fold respectively). In Gipz control cells, *Smoc2* expression was induced 3- to 3.5-fold upon 21 days of differentiation. We confirmed the effective reduction of secreted SMOC2 protein by Western blot analysis on supernatants of differentiated Sh*Smoc2* cells (Fig 2B). We found no significant differences in ALP activity between Gipz and Sh*Smoc2* cells at D1 and D21 (Fig 2C). Likewise, alizarin red staining did not differ between Gipz and Sh*Smoc2* cells upon differentiation (Fig 2D and 2E). These findings suggest that endogenous SMOC2 is not essential for osteogenic differentiation and ECM mineralization.

### The calcium binding domain partially mediates the inhibitory effects of SMOC2 on osteogenic differentiation and ECM mineralization

**Fig 3:**
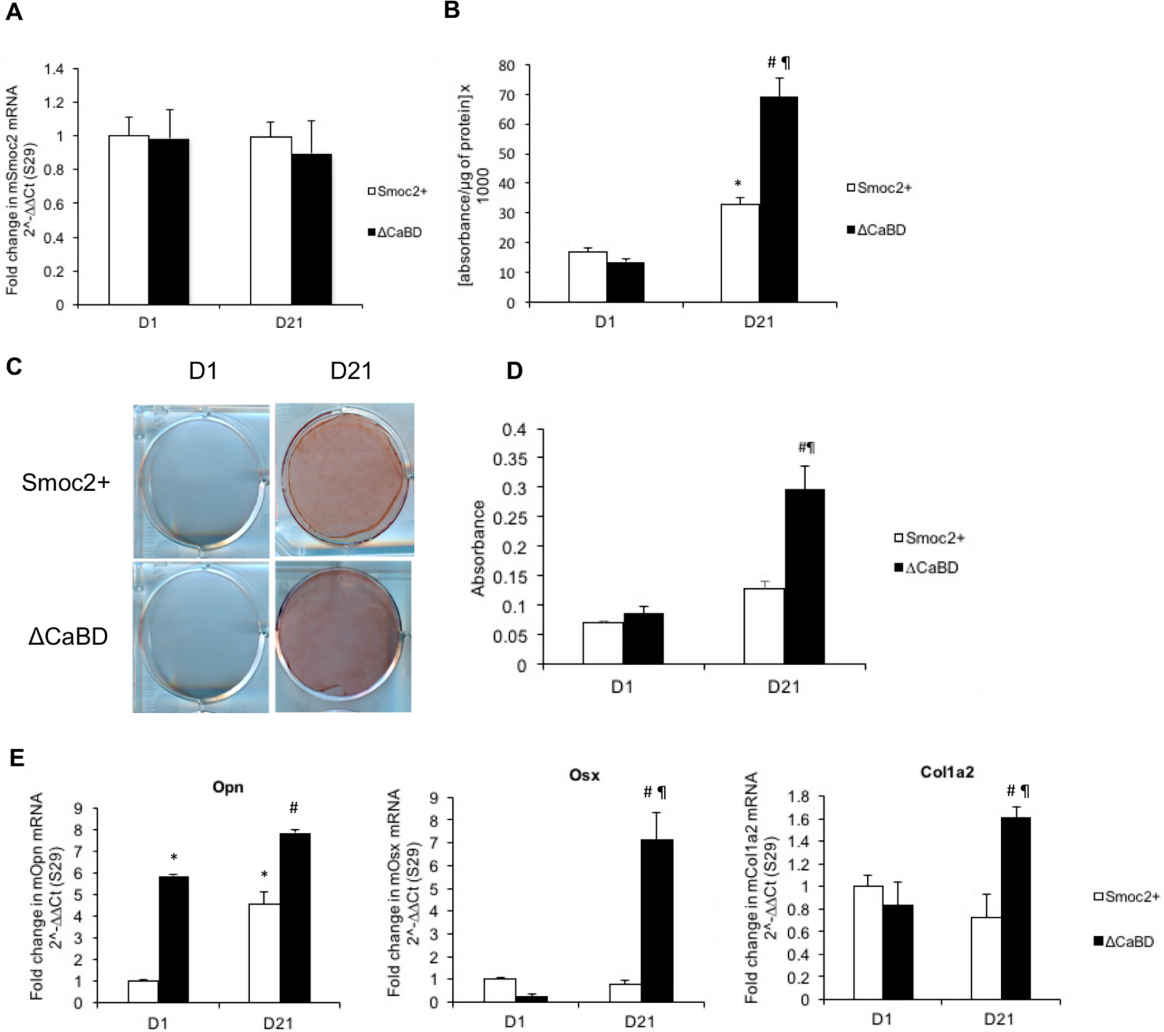
Influence of *Smoc2* ΔCaBD on differentiation and mineralization of MC3T3-E1 cells. Gene expression analysis of MC3T3-E1 cells overexpressing *Smoc2* lacking the calcium binding domain shows successful overexpression (A). At D21 increase in ALP activity (B) is higher and Alizarin red staining (C-D) is stronger in ΔCaBD cells compared to *Smoc2+*. (E) mRNA expression of *Osx*, *Opn*, *Col1a2* shows that ΔCaBD can decrease or suppress the influence of *Smoc2* overexpression on MC3T3-E1 differentiation. mRNA levels were normalized to *S29*. Statistically significant differences vs. D1 *Smoc2+* (internal control) are indicated as *: *P*<0.05; vs. ΔCaBD at D1 as ¶: *P*<0.05; vs. D21 *Smoc2+* as #: *P*<0.05). Figures are representative for one experiment with 3 different *Smoc2+* overexpressing clones performed in triplicate.

Calcium signaling plays an important role in osteogenic differentiation. We therefore studied whether the effects of SMOC2 on MC3T3-E1 cells were mediated by its calcium binding domain. To address this question, we generated MC3T3-E1 cells overexpressing mutant *Smoc2* lacking the calcium binding domain (ΔCaBD) and we compared them to MC3T3-E1 cells overexpressing full length *Smoc2* (*Smoc2+*). Fig 3A demonstrates successful and comparable overexpression of *Smoc2 ΔCaBD* and full length *Smoc*2 based on their relative mRNA levels. ALP activity and Alizarin red staining (Fig 3B–3D) of the cells did not differ at baseline. Following the 21 days differentiation protocol we detected a significant increase in ALP activity (2-fold) in differentiated ΔCaBD cells compared to *Smoc2+* cells (Fig 3B). Likewise, after 21 days of differentiation, ΔCaBD cells showed stronger mineralization compared to *Smoc2+* cells (Fig 3C and 3D). Gene expression analysis of osteoblast phenotype markers *Opn*, *Osx*, and *Col1a2* is depicted in Fig 3E. At D1, no significant differences could be detected in mRNA expression levels of *Osx* and *Col1a2* whereas *Opn* was significantly increased in ΔCaBD cells compared to *Smoc2+* cells. The increase in mRNA levels of *Opn*, *Osx* and *Col1a2* after 21 days of differentiation was significantly higher in ΔCaBD cells compared to *Smoc2+* cells. Taken together, these results indicate that the calcium binding domain of SMOC2 is playing a role in the inhibitory effects on osteogenic differentiation and ECM mineralization in MC3T3-E1 cells.

### Extracellular calcium supplementation diminishes the inhibitory effect of SMOC2 on osteogenic differentiation and ECM mineralization

**Fig 4:**
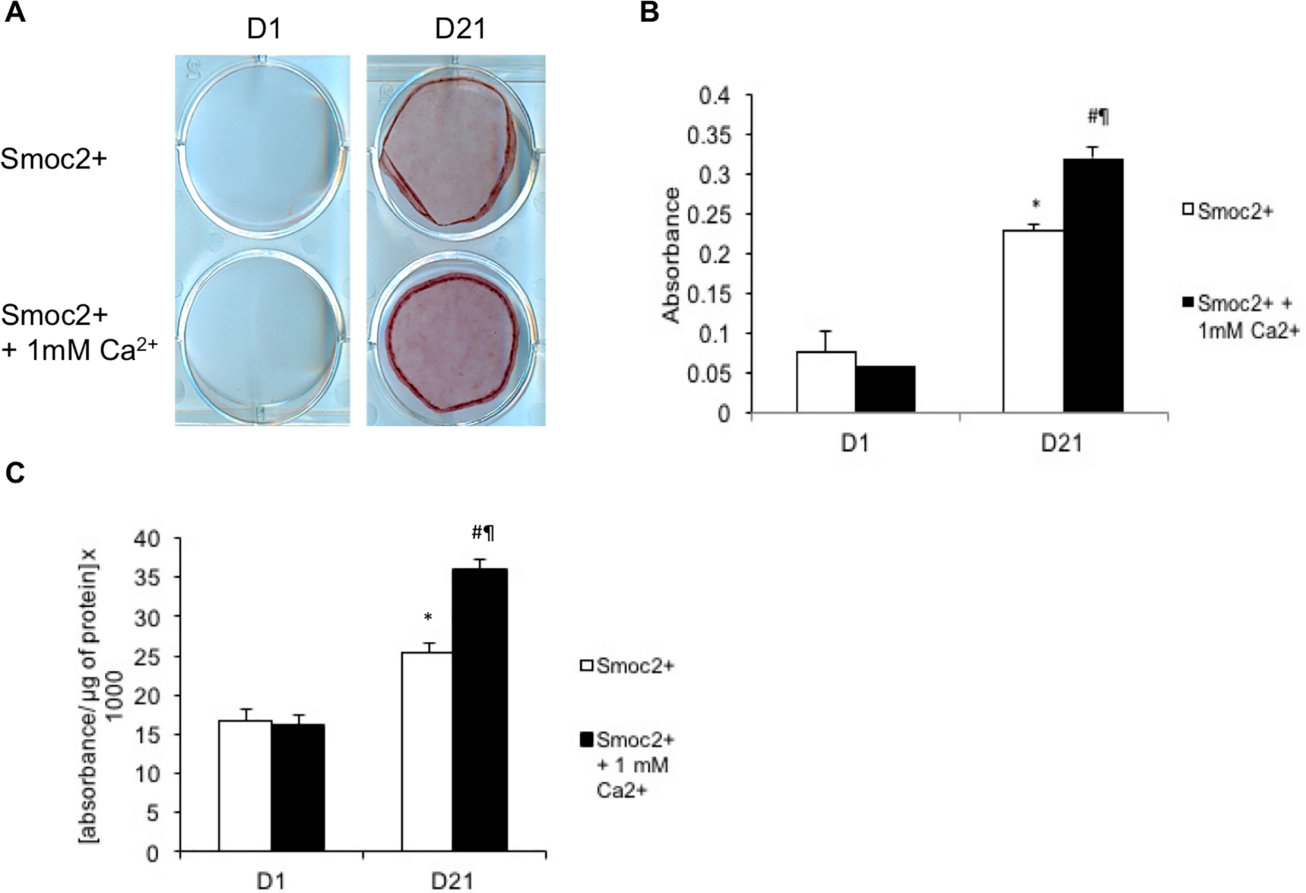
Influence of calcium supplementation to *Smoc2+* MC3T3-E1 cells on differentiation and mineralization. Addition of extracellular calcium to *Smoc2+* cells shows increased Alizarin Red staining (A-B) and a higher increase in ALP activity (C) at D21. Statistically significant differences vs. D1 *Smoc2+* (internal control) are indicated as *: *P*<0.05; vs. *Smoc2+* + 1mM Ca at D1 as ¶: *P*<0.05; vs. D21 *Smoc2+* as #: *P*<0.05). Figures are representative for one experiment with 2 different *Smoc2+* overexpressing clones performed in triplicate.

We hypothesized that high levels of SMOC2 could titer part of the calcium out of the extracellular environment, by capturing it through its calcium binding domain. To test this hypothesis, we evaluated whether the addition of extracellular calcium (1 mM of CaCl_2_) to the medium (2mM of CaCl_2_) during differentiation could rescue the impaired mineralization observed in *Smoc2+* cells. Therefore MC3T3-E1 *Smoc2+* cells were differentiated for 21 days in the absence or presence of extracellular calcium supplementation. Fig 4A shows no difference in alizarin red staining between undifferentiated *Smoc2+* and *Smoc2+* + 1mM CaCl_2_. However, at D21, *Smoc2+* + 1mM CaCl_2_ cells exhibited a stronger alizarin red staining than *Smoc2+* cells. This result suggests a rescue of mineralization in *Smoc2+* cells when extracellular calcium is added. Extracellular calcium addition also enhanced ALP activity in *Smoc2+* cells (Fig 4B). These data, in agreement with the results obtained with the calcium binding domain deletion, support that calcium plays a role in the impairment of SMOC2 on osteogenic differentiation and mineralization.

### SMOC2 impairs mineralization in primary human osteoblast precursors and of transdifferentiated HUVECs

**Fig 5:**
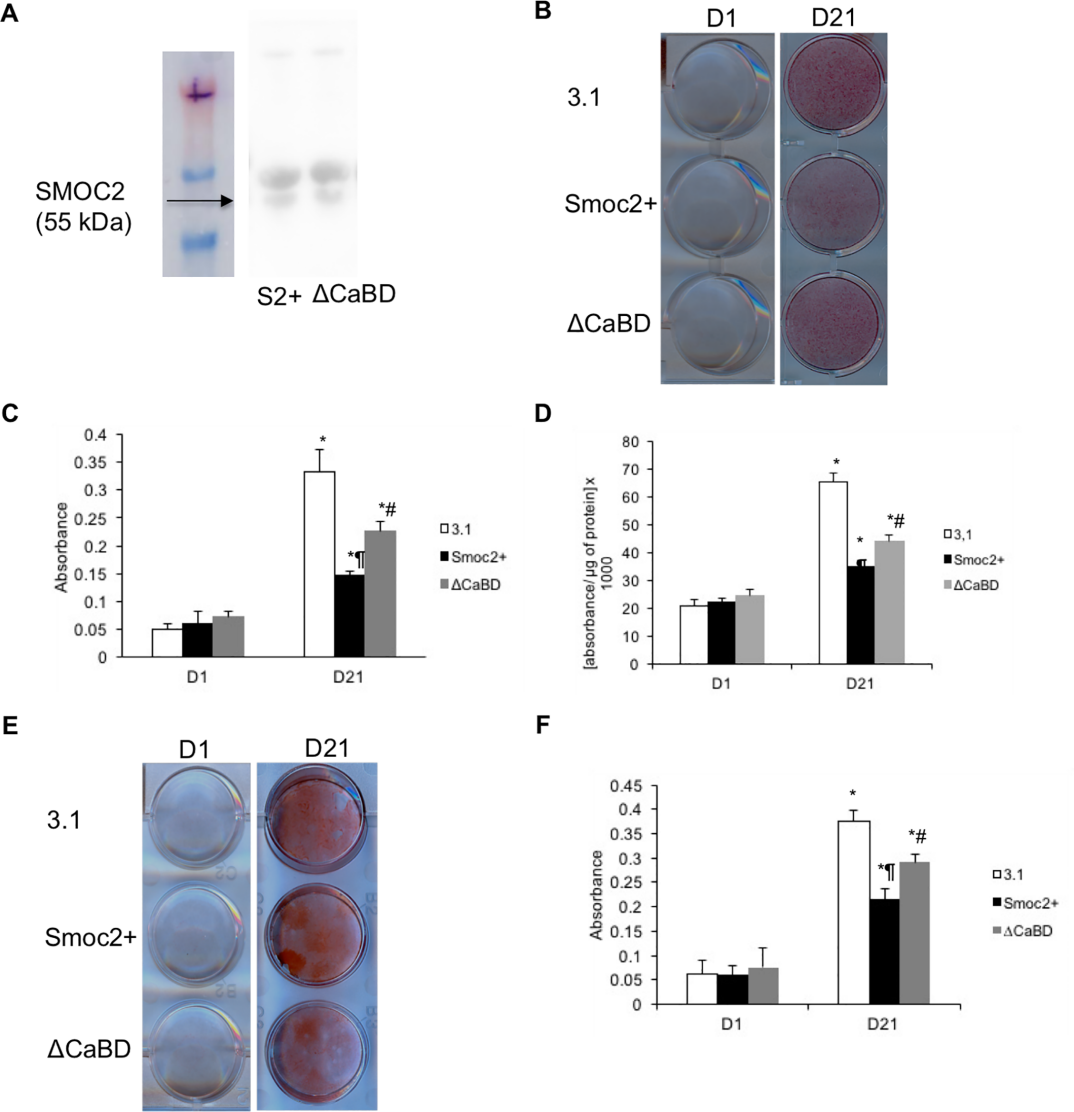
Influence of *Smoc2+* and of *Smoc2* ΔCaBD on differentiation and mineralization of hPDCs and HUVECs. (A) Confirmation of equal protein levels of secreted full length SMOC2 and SMOC2 ΔCaBD. (B-C) hPDCs treated with *Smoc2+* supernatant show less Alizarin staining compared to 3.1, while ΔCaBD produces an intermediate response. Similar results were obtained for ALP activity (D). (E) HUVECs treated with *Smoc2*+ supernatant show less Alizarin staining compared to 3.1, ΔCaBD being intermediate as demonstrated by quantification of absorbance at 405 nm (F). Statistically significant differences vs. D1 (within same condition) are indicated as *: *P*<0.05; vs. 3.1 at D21 as ¶: *P*<0.05; vs. D21 *Smoc2+* as #: *P*<0.05). Figures are representative of two independent experiments, performed in triplicate in hPDCs and of one experiment, performed in triplicate in HUVECs.

To translationally validate the data obtained in a murine cell line, we evaluated the influence of SMOC2 in a human *in vitro* model of osteogenesis. As SMOC2 is a secreted protein, we differentiated human periosteum derived mesenchymal cells for 21 days in the absence or presence of supernatants from MC3T3-E1 3.1, *Smoc2+* or ΔCaBD cells. Fig 5A confirms equal protein levels of secreted full length SMOC2 and SMOC2 ΔCaBD. At D21, Fig 5B and 5C and 5D show reduced alizarin red staining and ALP activity, respectively in the presence of *Smoc2+* supernatants compared to 3.1 controls. Notably, hPDCs in the presence of ΔCaBD supernatants exhibited a smaller reduction in ALP activity and alizarin red staining compared to *Smoc2+*. To further investigate the potential clinical implications of our findings, we explored whether SMOC2 could also have an impact on vascular calcification, which is a common complication of vascular diseases such as atherosclerosis, predicting cardiovascular morbidity and mortality [15–16]. To this end, we differentiated HUVECs for 21 days in the absence or presence of supernatants from MC3T3-E1 3.1, *Smoc2+* or ΔCaBD cells. After 21 days of differentiation, HUVECs cultured in the presence of *Smoc2+* supernatants exhibited a reduced alizarin red staining as compared to 3.1 control cells. Moreover, HUVECs in the presence of ΔCaBD supernatants exhibited a smaller decrease in Alizarin red staining compared to *Smoc2+* (Fig 5E and 5F). These results show that, consistent with our results in MC3T3-E1 cells, SMOC2 negatively modulates osteogenic differentiation and mineralization in primary human osteoblast precursors and in human endothelial cells. This suggests that SMOC2 could have a potential therapeutic benefit in diseases which involve vascular calcification such as atherosclerosis.

## Discussion

In this study, we investigated the influence of SMOC2 on mineralization and calcification in different model systems. First, we assessed the influence of SMOC2 on mineralization in the MC3T3-E1 cell line. Therefore, we used MC3T3-E1 cells overexpressing *Smoc2* or with *Smoc2* knockdown. Measurement of endogenous *Smoc2* level in control cells (both 3.1 and Gipz) shows *Smoc2* upregulation during the differentiation and mineralization process, which is however significantly lower than *Smoc2* levels in *Smoc2* overexpression cells. Cells overexpressing *Smoc2* show a severely reduced mineralization in this model. On the other hand, *Smoc2* knockdown did not affect mineralization. As endogenous *Smoc2* knockdown did not affect the mineralization process, we suggest the endogenous *Smoc2* upregulation in the differentiating cells might be a consequence of mineralization rather than a regulator and that the levels are too low to effectively inhibit mineralization. It has been described however that SMOC2 deficiency is associated with human and zebrafish dental and craniofacial defects [17–18]. Bloch-Zupan et al identified a homozygous *SMOC2* mutation in patients suffering from dentin dysplasia phenotype with major microdontia, oligodontia, and shape abnormalities. In a zebrafish study using the morpholino technique to knockdown *Smoc2*, they demonstrated abnormalities in the pharyngeal teeth similar to the human phenotype [17]. Melvin et al at the other hand identified *Smoc2* as a gene involved in craniofacial morphogenesis as *Smoc2* knockdown in Zebrafish led to craniofacial abnormalities [18]. A possible explanation for the contrast with our data is that MC3T3-E1 cells are derived from neonatal mouse calvaria, when cells are already committed to osteogenic differentiation [19]. Thus, the presence of *Smoc2* may be critical in earlier developmental stages.

Since calcium signaling plays an important role in osteogenic differentiation, we investigated the role of the EC domain in the inhibitory effects of SMOC2 [20–21]. Our results suggest that the inhibitory effect of SMOC2 is, at least in part, dependent on the EC domain, as a deletion of this domain partially rescues the inhibitory effects of wild type SMOC2 overexpression. We hypothesized that high levels of SMOC2 could titer part of the calcium out of the extracellular environment, by sequestering it through its calcium-binding domain. To test this hypothesis, we tried to measure calcium levels in conditioned medium of control cells, *Smoc2+* cells and ΔCaBD cells but could not demonstrate consistent differences. Different factors may explain this issue: (1) binding of SMOC2 and calcium is likely reversible as described by Novinec M et al [22], and the processing of the samples may affect this process. (2) Effects of SMOC2 may be linked to the actions close to the cell surface where matrix molecules are produced. Nevertheless, our hypothesis was supported by the phenotypic partial rescue upon extracellular calcium addition to SMOC2 overexpressing cells. Our observations on the overexpression of wild-type and truncated SMOC2 forms were replicated in a human model system, using hPDCs.

In this study, we focused on the calcium signaling as contributing factor to the inhibitory effects of SMOC2 on mineralization that we observed. However, as we only observe partial rescue effects with the calcium studies and based on literature, we acknowledge that the other domains of SMOC2 and thus other signaling pathways might also play a role in the observed effects. Moos et al showed in gain-of-function assays that XSMOC-1, the orthologue of human SMOC1, inhibited the BMP signaling as potently as known BMP antagonists. In the gain-of-function studies, they found that unlike known BMP antagonists such as noggin, XSMOC-1 antagonized BMP activity by acting downstream of the receptor. Loss-of-function studies using morpholinos, led to developmental failure indicating that XSMOC-1 is necessary for postgastrulation development [11]. Moos et al demonstrated moreover, using SMOC deletion constructs, that SMOC can both act as an expander or antagonist of BMP signaling, as SMOC-ΔEC, lacking the calcium-binding (EC) domain, inhibited BMP2 signaling whereas SMOC-EC (EC domain only) enhanced BMP signaling by expanding the range of signaling [23]. Mommaerts et al also demonstrated modulation of BMP target genes by SMOC2 in Zebrafish [24]. Altogether, their results suggest that the SMOC pathway could be a potential therapeutic target in diseases such as osteoarthritis, which are associated with an altered BMP signaling and altered calcification.

To investigate whether our results could have potential clinical implications, we explored the possible impact of SMOC2 on vascular calcification, which is a common complication of frequent vascular diseases such as atherosclerosis, predicting cardiovascular morbidity and mortality [15–16]. Therefore, we studied the effect of SMOC2 on HUVECs. Culturing HUVECs in the presence of *Smoc2+* supernatant caused decreased mineralization partially rescued when cultured in the presence of the EC-domain deletion mutant supernatant. These results suggest that SMOC2 could have beneficial effects in vascular calcification disorders.

In conclusion, we showed that SMOC2 overexpression inhibits mineralization in different model systems and identified the importance of the EC domain in this process.

## Supporting information

S1 Fig Cloning strategy to generate *Smoc2* ΔCaBD plasmid. The calcium-binding domain spans from aminoacid 352 to 412. For the first PCR reaction, the pcDNA3.1 plasmid containing wild type *Smoc2* and primer pair A (P1 and P2) to obtain the PCR product A and primer pair B (P3 and P4) to obtain PCR product B was used. PCR product A and B were used as templates and primers P1 and P4 were used in PCR reaction 2. The resulting product was the pcDNA3.1 plasmid containing mutant Smoc2 lacking the calcium binding domain (ΔAA352-412).
(JPG)

S1 Supplemental file The file contains the individual data points of the experiments reported in this article. (XLSX)
