# Supplementary figures and images for: SMOC2 inhibits calcification of osteoprogenitor and endothelial cells
Source: PLoS One. 2018 Jun 13;13(6):e0198104. doi: 10.1371/journal.pone.0198104 (PMC5999237; doi:10.1371/journal.pone.0198104)

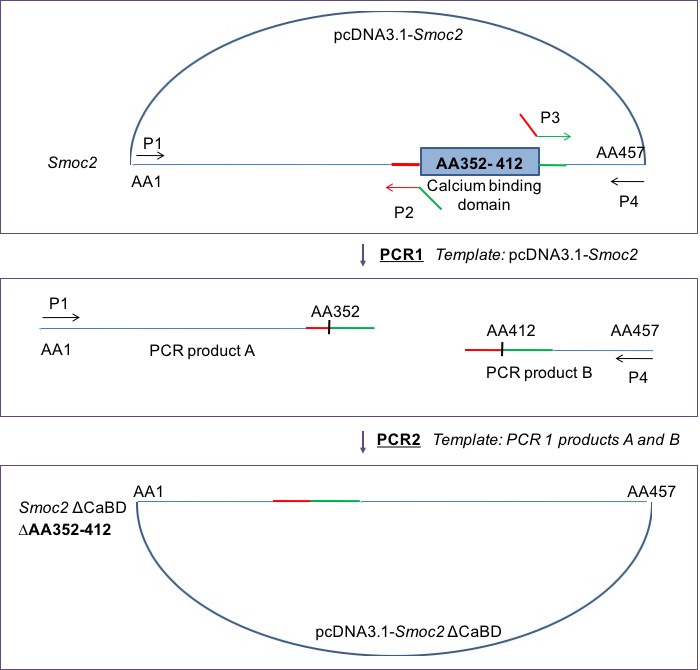

Supplement: S1 Fig — The calcium-binding domain spans from aminoacid 352 to 412. For the first PCR reaction, the pcDNA3.1 plasmid containing wild type Smoc2 and primer pair A (P1 and P2) to obtain the PCR product A and primer pair B (P3 and P4) to obtain PCR product B was used. PCR product A and B were used as templates and primers P1 and P4 were used in PCR reaction 2. The resulting product was the pcDNA3.1 plasmid containing mutant Smoc2 lacking the calcium binding domain (ΔAA352-412). (JPG) [file pone.0198104.s001.jpg]
